# Physiological Regulation of Pulmonary Microcirculation under Mechanical Ventilation at Different Cardiac Outputs and Positive End-Expiratory Pressures in a Porcine Model

**DOI:** 10.3390/jpm13010107

**Published:** 2023-01-03

**Authors:** Pan Pan, Lina Li, Fei Xie, Xingshuo Hu, Yinghua Guo, Lixin Xie, Longxiang Su

**Affiliations:** 1 College of Pulmonary and Critical Care Medicine Chinese PLA General Hospital, Beijing 100089, China; 2 Department of Critical Care Medicine, State Key Laboratory of Complex Severe and Rare Diseases, Peking Union Medical College Hospital, Chinese Academy of Medical Science and Peking Union Medical College, 1st Shuaifuyuan, Dongcheng District, Beijing 100730, China

**Keywords:** mechanical ventilation, pulmonary microcirculation, PEEP, shunt, transpulmonary capillary wall pressure (Pcap)

## Abstract

This study was performed to visualize the hemodynamic effects of pulmonary microcirculation and ventilation/perfusion (V/Q) matching after mechanical ventilation under different cardiac outputs and positive end-expiratory pressures (PEEPs). Ten experimental pigs were randomly divided into high and low tidal volume groups, and ventilation/perfusion were measured by electrical impedance tomography (EIT) at different PEEPs. Then, all the pigs were redivided into high cardiac output (CO) and low CO groups and measured by EIT at different PEEP levels with a low tidal volume. Additionally, sidestream dark field (SDF) was used to measure pulmonary microcirculation. Hemodynamic parameters and respiratory mechanics parameters were recorded. As PEEP increased at high tidal volume, blood flow was impaired at a higher PEEP (20 cmH_2_O) compared with low tidal volume (shunt: 30.01 ± 0.69% vs. 17.95 ± 0.72%; V/Q ratio: 65.12 ± 1.97% vs. 76.57 ± 1.25%, *p <* 0.01). Low tidal volume combined with an appropriate PEEP is the best option from the match between ventilation and pulmonary blood flow. Increasing PEEP can solve the problem of excessive shunt at high CO, and the V/Q ratio tends to match. At low CO, the increased dead space can reach as high as 64.64 ± 7.13% when PEEP = 20 cmH_2_O. With increasing PEEP, the microcirculation index deteriorates, including total vessel density (TVD), proportion of perfused vessel (PPV), perfused vessel density (PVD), and microcirculatory flow index (MFI). The periodic collapse of pulmonary capillaries or interruption of blood flow obviously occurred with high PEEP. The hemodynamic parameters indicated that the transpulmonary capillary wall pressure (Pcap) of the low CO group was negative at PEEP = 5 cmH_2_O, which determines the opening and closing of the pulmonary microcirculation and controls lung perfusion and the production of extravascular lung water. Therefore, it is essential to couple macrocirculation and pulmonary microcirculation during mechanical ventilation by improving shunting and optimizing Pcap.

## 1. Introduction

A pulmonary shunt is a pathological condition that results when the alveoli of the lungs are perfused with blood as normal, but ventilation fails to supply the perfused region. Excessive cardiac output (CO) can cause intrapulmonary shunts [1] (Lynch et al., 1979). In addition, the pulmonary capillaries can be stretched due to the expansion or collapse of alveoli under mechanical ventilation, resulting in a change in pulmonary vascular resistance and eventually affecting pulmonary microcirculation perfusion [2] (Whittenberger et al., 1960). Positive end-expiratory pressure (PEEP) is the pressure in the lungs (alveolar pressure) above atmospheric pressure (the pressure outside of the body) that exists at the end of expiration, which can affect pulmonary shunt. Improper positive end-expiratory pressure and tidal volume on lung injury may induce alveolar instability and hemodynamic disorder [3] (Halter et al., 2007). Karbing et al. found that improved lung aeration following an increase in PEEP is not always consistent with reduced shunt and V/Q mismatch [4] (Karbing et al., 2020). Fluid treatment cannot fully restore pulmonary microcirculation despite alleviated macrocirculation dysfunction during high PEEP ventilation [5] (He et al., 2019). Therefore, pulmonary blood flow is determined by CO and affected by alveolar ventilation and expansion. The matching of proper blood flow and ventilation is the key to affecting gas exchange, which needs to be fully considered in clinical treatment.

The ventilation/perfusion (V/Q) ratio was defined as the ratio of the amount of air reaching the alveoli per minute to the amount of blood reaching the alveoli per minute. With the progress of technology, sidestream dark field (SDF) and electric impedance tomography (EIT) visualization technology can provide us with a method to observe the changes in pulmonary ventilation and pulmonary microvascular blood flow directly. In particular, these technologies provide a useful tool for the real-time and visualized evaluation of the V/Q ratio at the bedside.

In this study, SDF and EIT visualization technology were used to study the effects of CO and PEEP on pulmonary ventilation and blood flow under physiological conditions by mechanical ventilation. The aim was to clarify the pulmonary microcirculation perfusion performance when V/Q changed due to CO and PEEP, which may contribute to a better understanding and guide mechanical ventilation strategies at different critical pathophysiological stages. The explanation of these mechanisms provides a theoretical basis for the protection of pulmonary circulation under mechanical ventilation.

## 2. Materials and Methods

### 2.1. Patient and Public Involvement Statement

This study is an animal experiment that did not involve any patients.

### 2.2. Animal Preparation

The experiments described below were approved by the Animal Care and Use Committee of Chinese PLA General Hospital (No. SQ2020052). Ten healthy male pigs that weighed 22.45 ± 2.28 kg were provided by the Animal Facility of Lvyuanweiye (Number of Animal use permit SYXK2021-0002). They were anesthetized and paralyzed for endotracheal intubation and then mechanically ventilated in a supine position. The induction of anesthesia was performed using an anesthesia machine (Veter 100, Prunus, Shenzhen, China). Anesthesia was induced with intramuscular atropine (0.04 mg/kg), tiletamine–zolazepam (6 mg/kg), and sevoflurane (2 L/min) and maintained by continuous infusion of propofol (2 mg/kg/h), midazolam (0.4 mg/kg/h), and fentanyl (10 µg/kg/h). Oral endotracheal intubation (7 mm, #201111135X, COVIDIEN, USA) and placement of a nasogastric pressure monitoring tube set (#BK0613, CareFusion, Germany) were performed. Mechanical ventilation was initiated with AVEA (AVEA, USA). Ventilation was set as the volume control model using the following parameters: tidal volume (VT) = 6 mL/kg, frequency (f) = 15 per min, PEEP = 5 cmH_2_O, oxygen inhalation (FiO_2_) = 30%, and SpO_2_ ≥ 95%, maintained by monitoring tongue oxygen saturation.

A right internal jugular vein catheter (#1C31018501, Cetofix, Braun, Germany) was placed percutaneously using the sterile technique. A Swan–Ganz (#744F75, Edwards Laboratories, Santa Ana, CA, USA) catheter was placed into the left external jugular vein. Femoral artery PiCCO (#20BC03, Edwards Laboratories, Santa Ana, CA, USA) was catheterized through the right femoral artery and connected to a PiCCO Module (PiCCO plus^®^ system; Pulsion Medical Systems, Munich, Germany). Related hemodynamic parameters were obtained directly as detected by the instruments, such as systolic artery pressure (SAP), diastolic artery pressure (DAP), mean artery pressure (MAP), heart rate (HR), central venous pressure (CVP), pulmonary capillary wedge pressure (PCWP), global end-diastolic volume (GEDV), extravascular lung water (EVLW), stroke volume variation (SVV), pulse pressure variation (PPV), cardiac output (CO), stroke volume (SV), systemic vascular resistance (SVR), and pulmonary vascular resistance (PVR). The arterial blood gases were also measured, including arterial oxygen saturation (SaO_2_), oxygen partial pressure (PO_2_), and partial pressure of carbon dioxide (PCO_2_), using a GEM Premier 3000 gas analyzer (“Instrumental laboratory”, USA).

### 2.3. EIT Measurements

Ventilation and perfusion measurements were obtained with PulmoVista500 (Dräger Medical, Lübeck, Germany). An EIT belt with 16 surface electrodes was placed around the pig’s thorax. A bolus of 10 mL 10% NaCl was injected through the central venous catheter during a respiratory pause (≥8 s). Ventilation was paused via an end-expiratory hold maneuver with the ventilator in the intubated pigs. The maneuver was repeated a maximum of once in 30 min if significant tortuosity/interruption in the regional impedance–time curve was observed. EIT data analysis was performed using customized software developed with MATLAB (R2015a, MathWorks, Natick, MA, USA). The related detailed methods are described in previous studies [6,7] (He et al., 2020a; He et al., 2020b).

### 2.4. SDF Measurements

The probe used for SDF microscopy was inserted through a fixed hole in the chest wall in the right lower thoracic cavity to perform microcirculatory measurements. The thoracic cavity was relatively sealed during the examination. Videos of the subpleural pulmonary microcirculation were recorded at expiration hold periods (5–8 s). Through slight movements of the probe, microcirculatory images at three different locations were collected during each examination. At each time point, the subpleural pulmonary microcirculation was evaluated at three different locations through SDF imaging (Microscan, Microvision Medical, Amsterdam, The Netherlands). Three 5 s video clips were recorded at each time point. The video clips used for analysis underwent a quality-control test based on image resolution quality, clarity of the image, and elimination of pressure-induced artifacts. The Automated Vascular Analysis software package (AVA 3.0 Microscan, Microvision Medical, Amsterdam, The Netherlands) was used for the analysis following expert consensus [8] (Ince et al., 2018). The related detailed methods are described in our previous study [5] (He et al., 2019).

### 2.5. The Detection of Esophageal Pressure and the Measurement of Transcapillary Wall Pressure (Pcap)

We use esophageal manometry to measure intrathoracic pressure. A nasogastric pressure monitoring tube set (#BK0613, CareFusion, Germany) was connected to the AVEA mechanical ventilator (AVEA, USA). We measured esophageal pressure (intrathoracic pressure) using end-inspiratory and end-expiratory occlusion. According to the formula of transpulmonary pressure [9,10] (Ganter et al., 2006; West, 2000), we calculated transcapillary wall pressure (Pcap): Pcap = PCWP-intrathoracic pressure (esophageal pressure monitoring).

### 2.6. Experimental Design and Protocol

**Figure 1:**
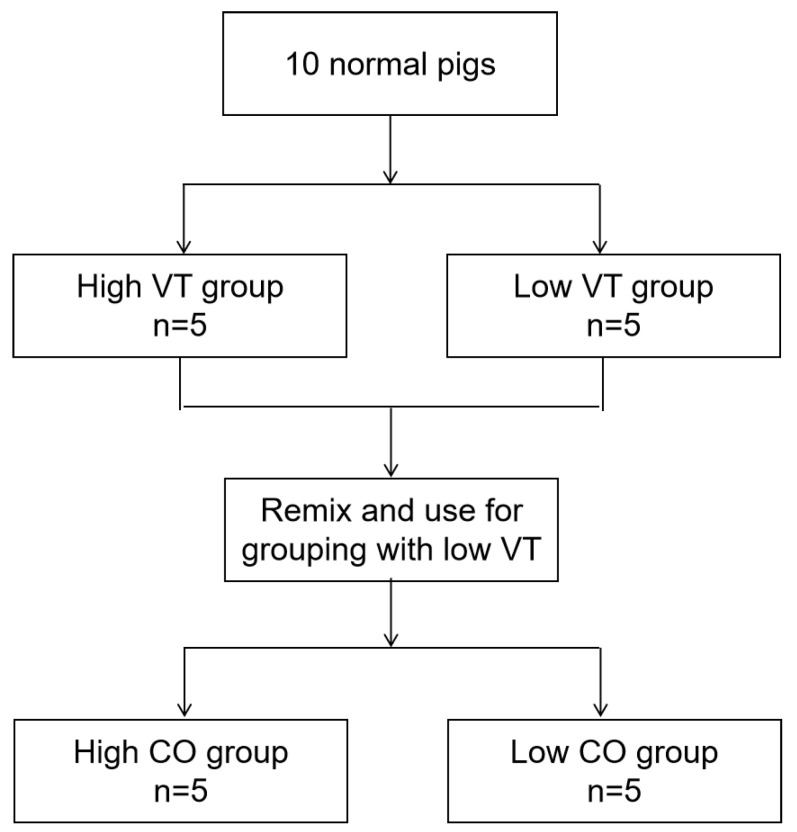
Flowchart of the experimental study. It should be noted that the adjustment of tidal volume is rapid in the initial stage. Relevant EIT tests were carried out immediately after the adjustment of tidal volume. All parameters were obtained within 30 min. It is reasonable to believe that this had little effect on the lung structure and final pathophysiological outcome in normal pigs. Of course, to further increase the rigor of this experiment, we randomized all pigs before CO grouping.

All 10 pigs were initially ventilated with lung protection with a small tidal volume (baseline VT = 6 mL/kg and PEEP = 5 cmH_2_O). First, we needed to determine the baseline tidal volume required for the study and the effect of PEEP on tidal volume. Five were randomly selected for baseline V/Q measurement by EIT and hemodynamic parameters (VT = 6 mL/kg and PEEP = 5 cmH_2_O). The other five used a larger tidal volume (VT = 15 mL/kg and PEEP = 5 cmH_2_O) to measure hemodynamic and respiratory mechanics and V/Q. EIT parameter detection under different tidal volumes was completed within 30 min. Second, we needed to explore the physiological effects of different COs and PEEPs. After the EIT measurement, the high tidal volume pigs returned to the baseline ventilation state for 30 min (VT = 6 mL/kg and PEEP = 5 cm H_2_O). Then, all the pigs were randomly redivided into two groups: a high cardiac output group (n = 5) and a low cardiac output group (n = 5). The flowchart is shown in Figure 1. The high cardiac output group received dobutamine 2 mL (20 mg) × 5 pcs + 10 mL saline via a pump in a 10–20 mL/h pipeline. CO rose to more than approximately 50% of the original by PiCCO. This high CO state was maintained for at least half an hour. The low cardiac output group adopted the bloodletting strategy to further reduce the CO, bleeding 200–400 mL until the CO dropped by 50% or more. This low CO state was maintained for at least half an hour.

PEEP started at 0 cmH_2_O and was adjusted up to 20 cmH_2_O and maintained for 10 min after each PEEP adjustment. Then, EIT measurements were performed at different PEEPs, including detection of ventilatory function and V/Q ratio. PEEP was set to 0, 5, 10, and 20 cmH_2_O, and each stage was monitored for approximately half an hour. At the same time, hemodynamic monitoring under the corresponding PEEP was carried out. Finally, the chest was opened for SDF measurement. At this time, the microcirculation parameters of the four stages of 0, 5, 10, and 20 cmH_2_O were recompleted. Here, we evaluated the hemodynamic effect of CO and PEEP rather than the final outcomes caused by such changes.

### 2.7. Statistical Analysis

The results for continuous variables with normal distributions are given as the means ± standard deviations (SDs). Student’s *t*-test was used to compare means between two groups. Analysis of variance (ANOVA) was used to compare means among multiple groups. The results for continuous variables that were not normally distributed are given as medians (25th and 75th percentiles) and were compared using nonparametric tests. The results for qualitative variables were expressed as percentages and compared between groups using a chi-square test. Statistical analyses were conducted by SPSS 16.0 (SPSS, Chicago, IL, USA), and a two-tailed *p <* 0.05 was considered significant.

## 3. Results

### 3.1. Changes in Lung Shunt, Dead Space, and Ventilation/Perfusion (V/Q) Ratio under the Influence of Tidal Volume and PEEP

**Figure 2:**
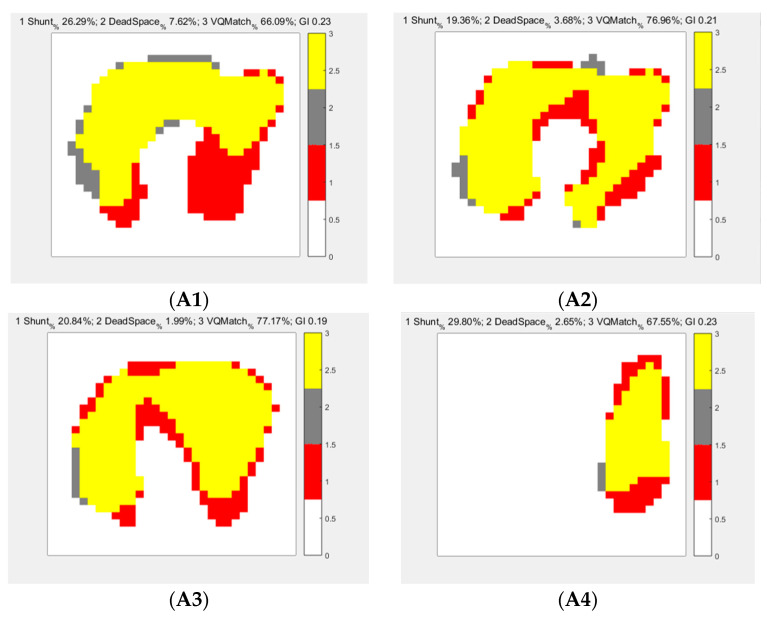

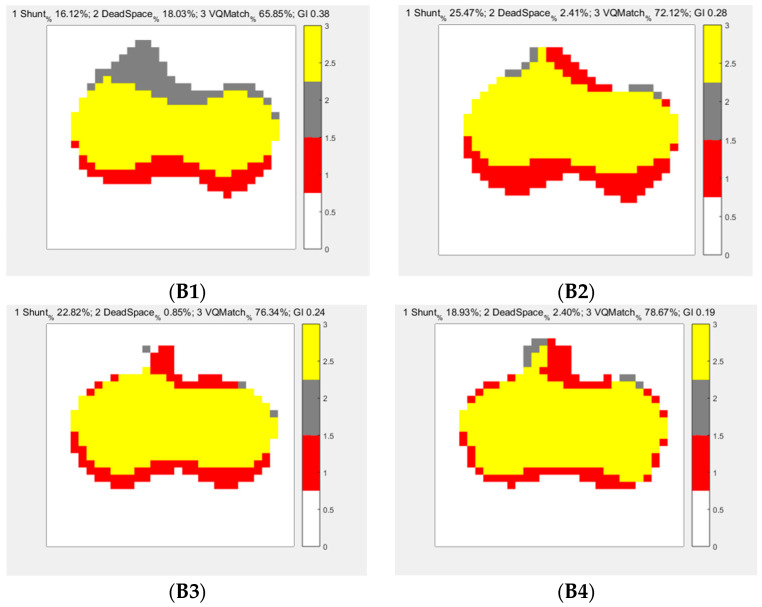
EIT images under different VTs and PEEPs. (**A**) High VT intervention (VT = 15 mL/kg), and (**B**) low VT intervention (VT = 6 mL/kg). The PEEP changes are shown from left to right of 0, 5, 10, and 20 cmH_2_O. During high VT with increasing PEEP, the shunt situation gradually improved, and the V/Q tended to be close to 0.8. However, the greater the PEEP was, the higher the peak airway pressure, and eventually, the right lung was not ventilated, which caused overshunting (30.01 ± 0.69%). During low VT with increasing PEEP, the dead space decreased, the shunt continued to decrease, and V/Q also tended to be close to 0.8. When PEEP = 20 cmH_2_O, the V/Q improvement of the low tidal volume group was more significant than that of the high tidal volume group (76.57 ± 1.25% vs. 65.12 ± 1.97%, *p <* 0.001). Please refer to the illustration on the right for different colors. Red stands for shunt, black stands for dead space, and yellow stands for VQ match.

Figure 2 and Table 1 show the data that were used to determine the high and low tidal volumes used in this study and explore the effect of different PEEPs on V/Q. When PEEP = 0 cmH_2_O, the high VT group showed an obvious shunt concentrated in the gravity-dependent area, and the low VT group showed an increase in the dead space of the nongravity-dependent area. Since this study is a physiological model of normal lungs, both groups showed improvement in shunting with a further increase in PEEP. Shunting improvement was manifested in the low VT group, showing that shunting in the gravity-dependent area decreased with increasing PEEP. However, in the high VT group, the shunt continued to increase. When PEEP = 20 cmH_2_O, the peak airway pressure was high and could not be ventilated. The V/Q ratio in the low VT group was higher than that in the high VT group at PEEP = 10 cmH_2_O and especially at 20 cmH_2_O with a statistically significant difference.

### 3.2. Changes in the Lung Ventilation/Perfusion (V/Q) Ratio under the Influence of High/Low Cardiac Output and Different PEEPs

**Figure 3:**
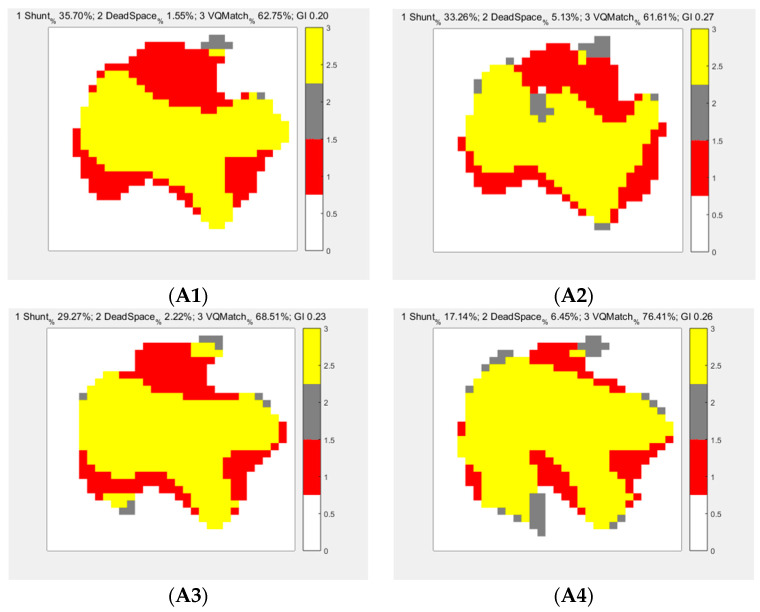

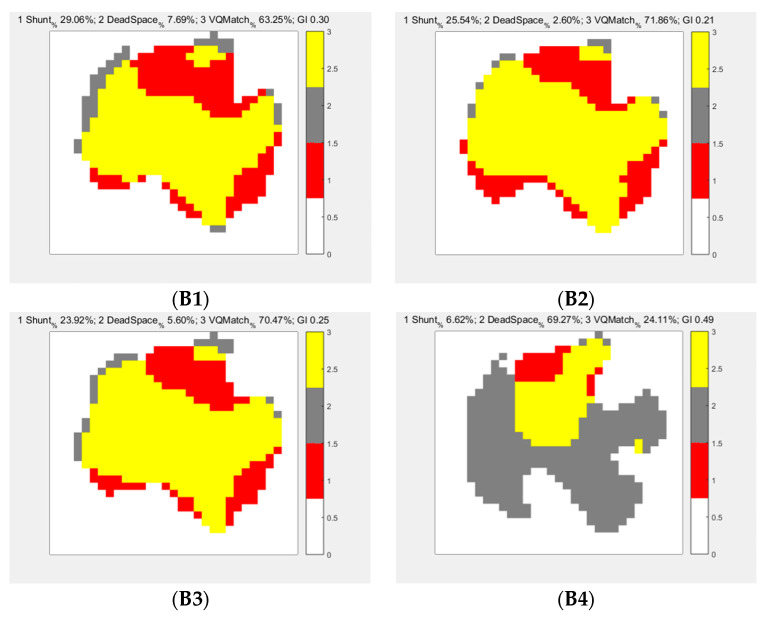
EIT images under high and low cardiac output (CO) and different PEEPs. (**A**) High flow intervention (high CO), and (**B**) low flow intervention (low CO). The PEEP changes are shown from left to right at 0, 5, 10, and 20 cmH_2_O. At high cardiac output, the shunt was large and improved with increased PEEP. At low cardiac output, the shunt showed limited improvement, and the dead space increased with increasing PEEP. The high cardiac output V/Q ratio improved obviously with increasing PEEP.

Changes in the lung ventilation/perfusion (V/Q) ratio and microcirculation under the influence of high/low CO and different PEEPs are shown in Table 2. During high CO (Figure 3A), as the PEEP increased, the shunt decreased, and the V/Q ratio tended to match. During low CO (Figure 3B), as the PEEP increased, the shunt tended to decrease, but the dead space tended to increase, which resulted in limited improvement. When PEEP = 20 cmH_2_O, the dead space was the main disadvantage (4.64 ± 7.13%). From the perspective of shunting, when PEEP = 0 cmH_2_O, the shunt of the high CO group (34.70 ± 1.63%) was significantly more obvious than that of the other two groups, and the difference was statistically significant. The shunt decreased with increasing PEEP, and when PEEP = 20 cmH_2_O, the degree of shunting of the high CO group was not significantly different from that of the baseline level. From the perspective of dead space, in the low CO group, the dead space increased with the increase in PEEP. When PEEP = 20 cmH_2_O, the dead space was as high as 4.64 ± 7.13%. The dead space of the baseline level was at its highest when PEEP = 0 cmH_2_O (16.53 ± 1.38%). Both the high CO and baseline levels showed relatively low dead space with increasing PEEP. From the V/Q ratio analysis, when PEEP = 0 cmH_2_O, PEEP = 5 cmH_2_O, and PEEP = 10 cmH_2_O, the V/Q ratio of the baseline level was better than that of the other two groups, but the V/Q ratio of the high CO group always improved with increasing PEEP. When PEEP =20 cmH_2_O, there was no difference in the V/Q ratio between the baseline level and the high CO group, but the V/Q ratio of the low CO group (27.29 ± 3.88%) was significantly reduced due to excessive dead space.

### 3.3. Changes in Pulmonary Microcirculation Perfusion under the Influence of High/Low Cardiac Output and Different PEEPs

**Figure 4:**
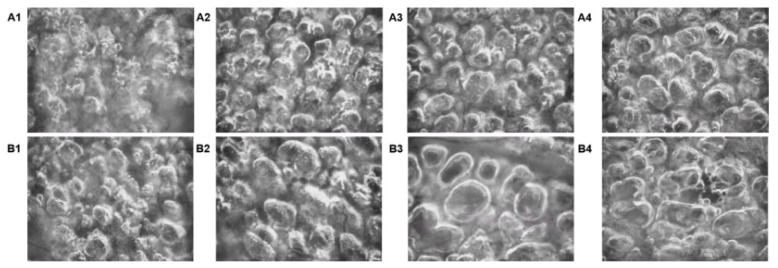
SDF images under high and low cardiac output and different PEEPs. At high cardiac output (Panel (**A**)), the microcirculation gradually improved with increasing PEEP. However, at low cardiac output (Panel (**B**)), with the increase in PEEP, the SDF showed more obvious vascular collapse and interruption of blood flow, which suggested local microcirculation occlusion. The video can be found in the Supplemental Video S2.

We used total vessel density (TVD), proportion of perfused vessels (PPV), perfused vessel density (PVD), and microvascular flow index (MFI) to evaluate pulmonary local microcirculation perfusion (Table 3). Overall, the microcirculation perfusion of the high CO group was better than that of the low CO group. At high CO, TVD gradually improved with increasing PEEP, but the change in TVD had no significant differences at PEEP values of 10 and 20 cmH_2_O. PVD, PPV, and MFI all tended to increase with higher PEEP. At low CO, when PEEP was 0 to 10 cmH_2_O, TVD did not change significantly, but TVD decreased significantly with a further increase in PEEP to 20 cmH_2_O. PPV and PVD improved when PEEP increased from 0 cmH_2_O to 10 cmH_2_O but decreased with a further increase in PEEP. TVD, PPV, and PVD decreased to their lowest values when PEEP was 20 cmH_2_O, and the difference was statistically significant compared with the high CO group. MFI improved when PEEP increased from 0 cmH_2_O to 5 cmH_2_O, but there was no statistical significance. However, with a further increase in PEEP, the MFI gradually decreased, and there was no blood flow or intermittent blood flow in a few vessels when PEEP = 20 cmH_2_O. SDF videos under high and low cardiac output and different PEEPs are provided in Figure 4 and Supplemental Video S1.

### 3.4. Hemodynamic Changes and Transpulmonary Capillary Wall Pressure (Pcap)

The changes in pulmonary and systemic hemodynamic parameters under different cardiac outputs and PEEPs are shown in Table 4. At high cardiac output, with the increase in PEEP, Pcap decreased, and extravascular lung water significantly decreased. At low cardiac output, Pcap was negative with increasing PEEP. The baseline level had the lowest Pcap and the lowest extravascular lung water. Accordingly, the oxygenation of the high CO group improved with increasing PEEP. The oxygenation of the low CO group worsened with increasing PEEP, and the oxygenation index was the highest at the baseline.

## 4. Discussion

First, we set high and low tidal volumes under normal cardiac output and directly observed the ventilation and blood flow of the lung under different PEEPs. The results showed the impairment of blood flow with an increase in PEEP at high tidal volume. Although the shunt and V/Q improved, the larger the PEEP was, the more difficult ventilation was. Additionally, we used the visualization technologies EIT and SDF to clarify lung ventilation and blood flow changes and the corresponding pulmonary microcirculation perfusion after different COs and different PEEPs under physiological conditions. We revealed that transpulmonary capillary wall pressure (Pcap) determines the opening and closing of the pulmonary microcirculation, controls lung perfusion, and produces extravascular lung water. These findings may guide clinicians to set mechanical ventilation parameters accurately with different COs and achieve lung and pulmonary microcirculation protection during mechanical ventilation.

The mechanism and consequences of ventilation-induced lung injury (VILI) are not airway pressure but lung hyperinflation [11] (Dreyfuss and Saumon, 1998). Our study found that the V/Q ratio changes under high tidal volume and that this change worsens with increasing PEEP. The reason is that the expansion of the lungs can affect pulmonary blood vessels [12] (Thomas et al., 1961). In the pulmonary interstitium, pulmonary microvessels can be lengthened due to the increase in pulmonary tidal volume. Since pulmonary microvessels are elastic tubes [13] (Guntheroth et al., 1982), the extension of the physiological range usually does not cause obvious stenosis of the microvessels. However, the pressure of intravascular pulmonary microcirculation is very low, and high tidal volume during mechanical ventilation can often cause pulmonary capillaries to become narrow when stretched, affecting pulmonary blood flow and causing damage [14] (Culver and Butler, 1980). When we set the experimental pigs to low tidal volume ventilation, we found that as PEEP increased, the shunt was continuously reduced, and the V/Q continued to improve. This may be because nongravity-dependent areas dominate tidal lung recruitment, while gravity-dependent areas are not effectively recruited. Under high VT, there is more blood and less gas in gravity-dependent areas, while low VT can ensure lung ventilation in nongravity-dependent areas. However, it cannot cause changes in the functional residual capacity, resulting in more gas and less blood. However, the low VT groups showed that as PEEP increased from 0 cmH_2_O, the shunt decreased. At PEEP = 5 cmH_2_O, the dead space of the low VT group was significantly reduced, and the shunt of the high VT group was significantly reduced. This is in line with the ability of PEEP to improve shunting, and it also matches the current lung protection ventilation strategy with low tidal volume and high PEEP. From the perspective of blood flow in the lung, if the tidal volume is low and the PEEP is small (PEEP = 0 or 5 cmH_2_O), the pulmonary microcirculation may not be ideal. The pulmonary blood vessels tend to close when the lung volume is too small and decreases below the functional residual capacity (FRC). This phenomenon may be due to the active tension in the vessel wall [15] (Lopez-Muniz et al., 1968). Studies have also found that a lung volume that is too low can cause irregular pulmonary microvasculature and collapse, which increases pulmonary vascular resistance [16] (Goshy et al., 1979). Therefore, a high tidal volume may lead to lung injury, while matching the appropriate high PEEP at a low tidal volume is beneficial for pulmonary microcirculation protection.

At low tidal volumes, we observed the influence of different COs on the shunt in the lungs. We adjusted the CO through cardiotonic drugs and bloodletting experiments and found that the intrapulmonary shunt was larger when the CO was high, and the shunt was smaller when the CO was low. Whether it is a change in CO caused by drugs, infusion/blood loss, or mechanical ventilation, it has been found that CO is always positively correlated with an intrapulmonary shunt [1,17,18] (Dantzker et al., 1980; Lynch et al., 1979; Smith et al., 1974). Lutch and Murray proposed that the effect of PEEP on altering FRC and on a cardiac index precluded the analysis of the independent effect of CO changes on the shunt [19] (Lutch and Murray, 1972). In this study, we found that regardless of the level of PEEP, the CO and shunt had a positive linear correlation, which further supports the independent influence of CO on the shunt. However, whether from the perspective of mechanical ventilation or CO, positive pressure ventilation itself will cause changes in CO. With higher tidal volume or higher PEEP, the venous return will decrease. The increase in afterload ultimately results in a decrease in CO [20] (Pinsky, 2018). Therefore, we must consider the impact of shunts when performing mechanical ventilation for different COs and PEEPs.

The extravascular lung water (EVLW) at high CO is significantly more than that at low CO, consistent with our previous clinical study [21] (Pan et al., 2019). In this animal model, we conducted a physiological study to illustrate the effect of CO on EVLW independently. Broccard et al. found that excessive vascular flow could aggravate lung injury and pulmonary edema [22] (Broccard et al., 1998). Teboul et al. [23] (Jozwiak et al., 2015) suggested that EVLW increased with fluid volume expansion at the same permeability. A higher CO, which meant more fluid volume in the pulmonary vasculature that could enhance the hydrostatic pressure, resulted in a high EVLW [23] (Jozwiak et al., 2015). Ehrhart et al. and Hasinoff et al. increased CO by 500% and 100%, respectively, and obtained increased amounts of EVLW [24,25] (Ehrhart et al., 1994; Skaburskis et al., 1989). For the formation of pulmonary edema, hydrostatic pressure is a critical factor. It is the difference between capillary pressure and extravascular pressure, called transvascular wall pressure [10] (West, 2000). According to the formula of transpulmonary pressure [9,10] (Ganter et al., 2006; West, 2000), we calculated transcapillary wall pressure (Pcap): Pcap = PCWP-intrathoracic pressure (esophageal pressure monitoring). Our results showed that the pressure across the capillary wall in the high CO group was greater than that in the low CO group, suggesting that the flow is related to changes in pressure across the capillary wall and pulmonary edema formation. For pulmonary edema caused by high CO, our study suggests that high CO matching high PEEP can better reduce Pcap, thereby reducing lung edema. When PEEP was set to the highest value of 20 cmH_2_O, the Pcap and lung water of the high CO group were the lowest, and the pulmonary microcirculation and oxygenation were also improved significantly.

We found that although EVLW seemed less abundant at low CO, the microcirculation situation was worse than that in the high CO group. We surprisingly found that Pcap even had a negative value in the low CO group at higher PEEP. This negative value did not promote the water in the interstitium returning to the capillaries. However, as the capillaries were occluded, there was no blood flow seen on the SDF videos. Our results show that under physiological conditions of low CO and high PEEP, although it seems that excessive EVLW production can be avoided, it is harmful to pulmonary microcirculation. Especially, we saw that the periodic collapse of pulmonary capillaries or interruption of blood flow obviously occurred with high PEEP. We also reasonably speculate that for lower CO, when the ARDS matches the higher PEEP, it aggravates lung microcirculation damage and further causes the regulation of ventilation and perfusion imbalance. Therefore, avoiding PEEP values that are too high at low CO is better for pulmonary microcirculation. We should adjust the flow of the macrocirculation to meet the flow of the pulmonary local circulation rather than only using PEEP titration during ARDS. We need to avoid shunting dysfunction, especially stretch and shear injuries caused by periodic opening of local blood vessels in the lung.

Low tidal volume lung protection ventilation helps protect the blood flow of the pulmonary circulation. An increase in the appropriate level of PEEP can reduce the intrapulmonary shunt, reduce the pressure across the pulmonary capillary vessel wall, and avoid the formation of excessive EVLW under high CO. However, it is necessary to pay attention to the V/Q mismatch caused by the collapse of pulmonary capillaries under low CO. Therefore, it is necessary to fully consider matching the macrocirculation to the pulmonary local microcirculation during mechanical ventilation. Transpulmonary vessel wall pressure (Pcap) may become a potential indicator of pulmonary microcirculation protection for the individualized treatment of mechanical ventilation in ARDS. It may be possible to use this parameter to avoid pulmonary vascular and endothelial damage in the future.

Our study also has some limitations. Firstly, this is an animal experiment. We used a limited number of pigs to complete the hemodynamic effects of tidal volume, CO, and PEEP, especially the titration of the relationship between lung local ventilation and blood flow. There may be confounding effects of the different study conditions. However, what we studied is the change in normal lung under different respiratory mechanical conditions. On the basis of pathophysiological principles, we reasoned that this effect would not be greater than that in animal models of ARDS. Secondly, the main purpose of this study was to study the physiological effects of different COs and different PEEPs. Therefore, our study only illustrates the relationship between lung ventilation and V/Q under different conditions, rather than specify a clear study endpoint. Through this study, we can explore a possible execution condition for the next step to find an optimal ventilation scheme, and then proceed to the design and verification of randomized controlled animal experiments. SDF can only be measured at the end of the experiment as it requires invasive monitoring. In the future, it is hoped that there will be a method and device capable of continuous monitoring for real-time measurement of local pulmonary microcirculation.

## 5. Conclusions

SDF and EIT are bedside noninvasive technical means that can visualize and reflect the patient’s ventilation and blood flow status in real time. This study first visualized the matching degree of ventilation and perfusion (V/Q) and realized the perfusion of lung and pulmonary microcirculation. We define it as a phenomenon of periodic switching of pulmonary circulatory microvessels. Low tidal volume lung protection ventilation helps to protect the blood flow of the pulmonary circulation from ventilation and perfusion. An increase in the appropriate level of PEEP can reduce the intrapulmonary shunt, reduce the pressure across the pulmonary vessel wall, and avoid the formation of excessive lung water under high CO. However, the collapse of pulmonary capillaries under low CO caused uncoupled ventilation and perfusion. Transpulmonary vessel wall pressure (Pcap) should be considered in the regulation of lung ventilation and blood flow coupling, which may become a potential indicator of pulmonary circulation protection for the individualized treatment of mechanical ventilation in ARDS.

## Figures and Tables

**Table 1:** Changes in lung shunt, dead space, and ventilation/perfusion (V/Q) ratio under the influence of tidal volume and PEEP.

	PEEP Level (cmH_2_O)	Low VT Group	High VT Group	*p* Value
		VT = 6 mL/Kg, N = 5	VT = 15 mL/Kg, N = 5	
Shunt (%)	0	16.99 ± 1.25	25.58 ± 2.22	<0.01
	5	27.01 ± 1.19	20.17 ± 0.96	<0.01
	10	21.86 ± 1.15	26.40 ± 1.28	<0.01
	20	17.95 ± 0.72	30.01 ± 0.69	<0.01
Dead space (%)	0	16.53 ± 1.38	7.73 ± 1.12	<0.01
	5	3.26 ± 0.60	3.81 ± 0.53	0.166
	10	1.80 ± 0.73	2.31 ± 0.41	0.209
	20	1.70 ± 0.56	2.13 ± 0.38	0.187
V/Q ratio (%)	0	66.90 ± 1.85	63.35 ± 3.17	0.063
	5	74.67 ± 1.59	72.57 ± 3.11	0.216
	10	78.75 ± 1.51	75.45 ± 1.34	0.006
	20	76.57 ± 1.25	65.12 ± 1.97	<0.001

Quantitative data with a normal distribution are presented as the mean ± SD.

**Table 2:** Changes in lung shunt, dead space, and ventilation/perfusion (V/Q) ratio under the influence of high/low cardiac output and different PEEPs.

	PEEP Level (H_2_O)	Baseline	High CO Group	Low CO Group	*p* Value
		N = 10	N = 5	N = 5	
Shunt (%)	0	16.99 ± 1.25	34.70 ± 1.63 *	22.88 ± 4.04 *#	<0.001
	5	27.01 ± 1.19	30.88 ± 1.50 *	25.02 ± 1.83 #	<0.001
	10	21.86 ± 1.15	24.21 ± 0.99 *	21.05 ± 3.53 #	0.105
	20	17.95 ± 0.72	19.14 ± 1.52	4.20 ± 1.32 *#	<0.001
Dead space (%)	0	16.53 ± 1.38	2.42 ± 0.69 *	8.27 ± 1.08 *#	<0.001
	5	3.26 ± 0.60	6.99 ± 1.51 *	3.82 ± 1.31 #	0.001
	10	1.80 ± 0.73	3.52 ± 1.08 *	5.45 ± 1.30 *	0.001
	20	1.70 ± 0.56	4.66 ± 0.77 *	4.64 ± 7.13 *	<0.001
V/Q ratio (%)	0	66.90 ± 1.85	61.30 ± 1.96	55.62 ± 2.54 *	<0.001
	5	74.67 ± 1.59	70.06 ± 2.55	64.31 ± 2.45 *#	<0.001
	10	78.75 ± 1.51	74.11 ± 1.06	71.79 ± 1.70 *	<0.001
	20	76.57 ± 1.25	77.53 ± 1.62	27.29 ± 3.88 *#	<0.001

For the baseline mean, the shunt, dead space, and V/Q ratio were measured in all pigs before they were randomly grouped by CO. Quantitative data with a normal distribution are presented as the mean ± SD. * represents the comparison vs. baseline, *p* < 0.05; # represents the comparison vs. high CO group, *p* < 0.05.

**Table 3:** Physiological regulation mechanism of pulmonary microcirculation under mechanical ventilation at different cardiac outputs and PEEPs.

	PEEP Level (cmH_2_O)	Low CO Group	High CO Group	*p* Value
		N = 5	N = 5	
TVD	0	3.85 ± 0.44	4.12 ± 0.16	0.246
	5	3.95 ± 0.23	4.24 ± 0.18	0.056
	10	3.16 ± 0.15	4.55 ± 0.27	<0.001
	20	2.06 ± 0.43	4.74 ± 0.65	<0.001
PPV%	0	75.25 ± 4.01	82.81 ± 5.25	0.034
	5	76.02 ± 3.81	86.95 ± 2.97	0.001
	10	79.80 ± 4.91	91.11 ± 3.89	0.004
	20	64.92 ± 8.37	90.10 ± 5.57	0.001
PVD	0	2.02 ± 0.29	3.50 ± 1.93	<0.001
	5	2.28 ± 0.25	4.05 ± 0.17	<0.001
	10	2.75 ± 0.30	4.45 ± 0.51	0.002
	20	1.81 ± 0.31	4.55 ± 0.39	<0.001
MFI	0	1.22 ± 0.22	2.19 ± 0.22	<0.001
	5	1.50 ± 0.20	2.29 ± 0.26	0.001
	10	1.27 ± 0.25	2.57 ± 0.13	<0.001
	20	0.85 ± 0.28	2.62 ± 0.26	<0.001

TVD, total vessel density; PPV, proportion of perfused vessels; PVD, perfused vessel density; MFI, microvascular flow index. Quantitative data with a normal distribution are presented as the mean ± SD.

**Table 4:** Hemodynamic changes and transpulmonary capillary wall pressure.

	PEEP Level (cmH_2_O)	Baseline	High Flow Group	Low Flow Group	*p* Value
		N = 10	N = 5	N = 5	
CO (L/min)	0	2.99 ± 0.22	4.02 ± 0.29 *	1.71 ± 0.27 *#	<0.01
	5	2.77 ± 0.19	3.76 ± 0.38 *	1.56 ± 0.23 *#	<0.01
	10	2.51 ± 0.23	3.55 ± 0.41 *	1.37 ± 0.25 *#	<0.01
	20	2.29 ± 0.22	3.31 ± 0.41 *	1.20 ± 0.22 *#	<0.01
CVP (mmHg)	0	1.60 ± 0.55	4.20 ± 0.45 *	1.20 ± 0.45 *#	<0.01
	5	1.80 ± 0.45	4.40 ± 0.55 *	2.00 ± 0.71 #	<0.01
	10	2.20 ± 0.84	4.80 ± 0.45 *	2.60 ± 0.55 #	<0.01
	20	3.00 ± 0.71	5.80 ± 0.45 *	3.40 ± 0.55 #	<0.01
MAP (mmHg)	0	84.80 ± 3.96	86.00 ± 4.06	85.60 ± 3.44	0.882
	5	83.20 ± 4.09	85.40 ± 4.28	84.20 ± 3.96	0.706
	10	82.40 ± 3.58	84.00 ± 3.31	82.60 ± 5.18	0.802
	20	80.80 ± 4.43	82.60 ± 3.36	81.40 ± 5.59	0.82
mPAP (mmHg)	0	12.20 ± 0.84	16.60 ± 0.89 *	18.20 ± 0.84 *	<0.001
	5	12.60 ± 0.55	17.40 ± 0.89 *	19.20 ± 1.30 *	<0.001
	10	13.20 ± 0.10	19.40 ± 1.34 *	21.40 ± 1.14 *	<0.001
	20	13.60 ± 1.14	21.00 ± 0.71	22.80 ± 0.84 *	<0.001
PCWP (mmHg)	0	9.60 ± 0.55	10.80 ± 0.84 *	9.00 ± 0.71	0.005
	5	10.20 ± 0.84	12.40 ± 0.55 *	10.20 ± 0.84	0.01
	10	11.20 ± 0.84	13.40 ± 0.55 *	10.80 ± 0.84 #	<0.001
	20	11.80 ± 1.31	14.40 ± 0.55 *	11.60 ± 1.14 #	0.002
EVLWI (mL/kg)	0	9.30 ± 0.72	14.18 ± 0.75 *	9.42 ± 0.22 #	<0.001
	5	8.50 ± 0.83	12.96 ± 0.75 *	8.86 ± 0.35 #	<0.001
	10	8.06 ± 0.46	11.43 ± 0.86 *	8.04 ± 0.36 #	<0.001
	20	7.32 ± 0.41	10.14 ± 0.76 *	7.39 ± 0.32 #	<0.001
PVPI	0	2.09 ± 0.09	2.20 ± 0.19	2.18 ± 0.18	0.523
	5	2.12 ± 0.11	2.24 ± 0.17	2.19 ± 0.12	0.382
	10	2.11 ± 0.04	2.14 ± 0.20	2.27 ± 0.15	0.249
	20	2.21 ± 0.08	2.21 ± 0.21	2.23 ± 0.12	0.946
PESins (mmHg)	0	8.44 ± 0.74	9.02 ± 0.75	8.32 ± 0.61	0.279
	5	9.60 ± 0.74	10.80 ± 0.49 *	10.50 ± 0.74	0.037
	10	10.64 ± 0.69	11.78 ± 1.13	11.86 ± 0.69	0.08
	20	11.58 ± 1.29	12.91 ± 0.94	13.00 ± 0.89	0.095
Pcap (mmHg)	0	1.16 ± 0.39	1.78 ± 0.54 *	0.68 ± 0.16 *#	0.03
	5	0.60 ± 0.31	1.60 ± 0.61 *	−0.30 ± 0.31 *#	<0.001
	10	0.56 ± 0.23	1.62 ± 0.59 *	−0.16 ± 0.21 *#	<0.001
	20	0.22 ± 0.11	1.48 ± 0.50 *	−1.40 ± 0.30 *#	<0.001
PO_2_ (mmHg)	0	90.00 ± 9.25	85.60 ± 7.30	92.80 ± 8.14	0.409
	5	100.40 ± 12.56	92.80 ± 6.61	88.20 ± 8.53	0.168
	10	98.00 ± 12.83	110.20 ± 15.04 *	79.80 ± 9.31 *#	0.008
	20	85.60 ± 6.11	111.20 ± 14.65 *	66.40 ± 9.94 *#	<0.001
PO_2_/FiO_2_	0	300.00 ± 30.82	285.33 ± 24.34	309.33 ± 27.12	0.409
	5	334.67 ± 41.87	309.33 ± 22.04	294.00 ± 28.42	0.168
	10	326.67 ± 42.75	298.62 ± 28.47	159.60 ± 18.62 *#	<0.001
	20	285.33 ± 20.36	278.00 ± 36.63	132.80 ± 19.88 *#	<0.001

Quantitative data with a normal distribution are presented as the mean ± SD. * represents the comparison vs. baseline, *p* < 0.05; # represents the comparison vs. high CO group, *p* < 0.05.

## Data Availability

Not applicable.

## Supplementary Materials

Supplemental Video S1. SDF images under high cardiac output and different PEEPs. Panels A–D represent PEEPs from 0–20 cmH_2_O. A Su: Longxiang (2022): SV1A_High CO with PEEP = 0.mp4. figshare. Media. https://doi.org/10.6084/m9.figshare.20425266.v1 (accessed on 1 January 2022). B Su: Longxiang (2022): SV1B_High CO with PEEP = 5.mp4. figshare. Media. https://doi.org/10.6084/m9.figshare.20425278.v1. C Su, Longxiang (2022): SV1C_High CO with PEEP = 10. figshare. Media. https://doi.org/10.6084/m9.figshare.20425281.v1 (accessed on 1 January 2022). D Su: Longxiang (2022): SV1D_High CO with PEEP = 20. figshare. Media. https://doi.org/10.6084/m9.figshare.20425296.v1 (accessed on 1 January 2022). Supplemental Video S2. SDF images under low cardiac output and different PEEPs. Panels A–D represent PEEPs from 0–20 cmH_2_O. A Su: Longxiang (2022): SF2A_low CO with PEEP = 0. figshare. Media. https://doi.org/10.6084/m9.figshare.20425305.v1 (accessed on 1 January 2022). B Su: Longxiang (2022): SF2B_low CO with PEEP = 5. figshare. Media. https://doi.org/10.6084/m9.figshare.20425311.v1 (accessed on 1 January 2022). C Su: Longxiang (2022): SF2C_low CO with PEEP = 10. figshare. Media. https://doi.org/10.6084/m9.figshare.20425308.v1 (accessed on 1 January 2022). D Su: Longxiang (2022): SF2D_low CO with PEEP = 20. figshare. Media. https://doi.org/10.6084/m9.figshare.20425317.v1 (accessed on 1 January 2022).
